# A simple and cost-efficient technique to generate hyperpolarized long-lived ^15^N-^15^N nuclear spin order in a diazine by signal amplification by reversible exchange

**DOI:** 10.1063/1.5132308

**Published:** 2020-01-02

**Authors:** Soumya S. Roy, Peter J. Rayner, Michael J. Burns, Simon B. Duckett

**Affiliations:** 1 Centre for Hyperpolarisation in Magnetic Resonance (CHyM), Department of Chemistry, University of York, Heslington, York YO10 5DD, United Kingdom; 2 Department of Inorganic and Physical Chemistry, Indian Institute of Science, Bangalore 560012, India

## Abstract

Signal Amplification by Reversible Exchange (SABRE) is an inexpensive and simple hyperpolarization technique that is capable of boosting nuclear magnetic resonance sensitivity by several orders of magnitude. It utilizes the reversible binding of *para*-hydrogen, as hydride ligands, and a substrate of interest to a metal catalyst to allow for polarization transfer from *para*-hydrogen into substrate nuclear spins. While the resulting nuclear spin populations can be dramatically larger than those normally created, their lifetime sets a strict upper limit on the experimental timeframe. Consequently, short nuclear spin lifetimes are a challenge for hyperpolarized metabolic imaging. In this report, we demonstrate how both hyperpolarization and long nuclear spin lifetime can be simultaneously achieved in nitrogen-15 containing derivatives of pyridazine and phthalazine by SABRE. These substrates were chosen to reflect two distinct classes of ^15^N_2_-coupled species that differ according to their chemical symmetry and thereby achieve different nuclear spin lifetimes. The pyridazine derivative proves to exhibit a signal lifetime of ∼2.5 min and can be produced with a signal enhancement of ∼2700. In contrast, while the phthalazine derivative yields a superior 15 000-fold ^15^N signal enhancement at 11.7 T, it has a much shorter signal lifetime.

## I. INTRODUCTION

Despite the many significant advances that have taken place in Nuclear Magnetic Resonance (NMR) since its inception, poor sensitivity still limits full utility. This low sensitivity arises because NMR relies on the Boltzmann distribution to create population imbalances between the nuclear spin orientations it probes.^1^ While ^1^H detection offers maximum sensitivity, the signal amplitude still originates from a difference of just 1 in each 32 000 ^1^H spins at room temperature (RT) within a 9.4 T magnet.^1^ This problem is even more pronounced for low-γ nuclei such as ^13^C and ^15^N, where in the latter case, just 1 in every 300 000 ^15^N nuclear spins contributes positively at this field.^1,2^

Recent developments in hyperpolarization techniques that improve sensitivity have allowed the development of magnetic resonance applications that were previously thought to be beyond the techniques’ reach.^3^ This builds from the fact that techniques such as Dynamic Nuclear Polarization (DNP)^4^ and Spin Exchange Optical Pumping (SEOP)^5^ provide unprecedented levels of signal enhancement for carbon-13, nitrogen-15, and xenon-129 spin detection. While these developments have been applied to the *in vivo* study,^6–8^ they often involve high-cost instrumentation^4^ which acts to restrict their utilization.

**SCHEME 1.**
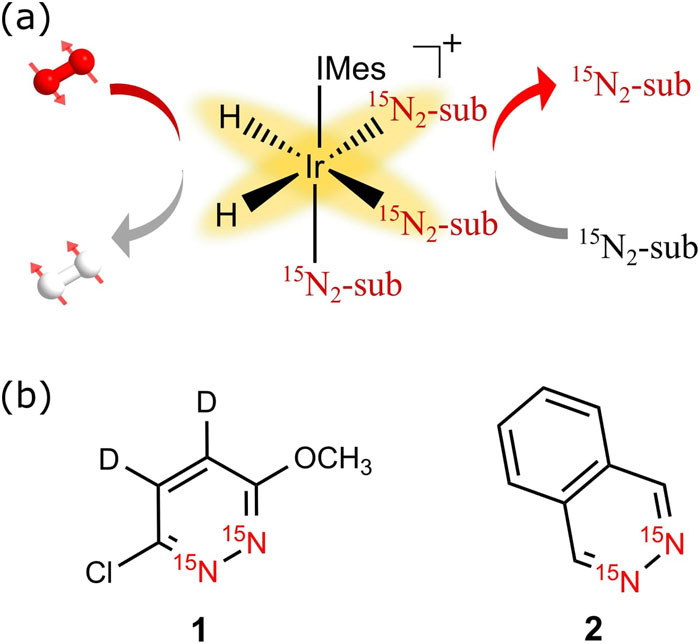
(a) Schematic depiction of the SABRE hyperpolarization method; *p*-H_2_ and substrate (sub) bind reversibly to an iridium catalyst to induce polarization transfer. (b) Structures of the substrates used in this study—3-chloro-6-methoxy-4,5-*d*_2_-pyridazine-^15^N_2_ (**1**) and phthalazine-^15^N_2_ (**2**).

An alternative approach involving *para*-hydrogen (*p*-H_2_) as a source of polarization is gaining popularity due to its speed and simplicity.^9,10^ Methods involving *p*-H_2_ are referred to as *Para*-Hydrogen Induced Polarization (PHIP) approaches and classically use a metal catalyst to add *p*-H_2_ to an unsaturated substrate via a hydrogenation step. However, a variant of PHIP called Signal Amplification by Reversible Exchange (SABRE) has greatly expanded the remit of the PHIP method as it does not induce chemical change in the substrate.^11^ SABRE instead employs reversible substrate and *p*-H_2_ binding to a catalyst to transfer polarization from the *p*-H_2_ derived hydride ligands into a selected substrate under appropriate resonance conditions (Scheme 1).^12^ Since its inception, SABRE has become successful at hyperpolarizing a growing range of important materials such as nicotinamide, methyl nicotinate, imidazole, diazirines, metronidazole, amines, and pyruvate.^13–20^

The hyperpolarization of heteronuclei provides two crucial advantages over normal ^1^H magnetic resonance imaging (MRI)—(a) an essentially background-free signal and (b) potentially long magnetic state lifetimes. This is reflected in the fact that the greatest success of DNP to date has been the hyperpolarization of ^13^C nuclei in isotopically labeled pyruvate for the subsequent study of metabolic pathways linked to cancer.^6,21–23^ Hyperpolarized ^15^N offers similar advantages to ^13^C detection, and the feasibility of its use *in vivo* has been established previously for ^15^N-choline.^24^ As the relative molar receptivity of ^15^N is just $1.04\times 10^{-3}$ and ^13^C $1.59\times 10^{-2}$ with respect to ^1^H, the use of hyperpolarization is critical for such heteronucleus detection.^1^

Warren and co-workers have demonstrated that ^15^N targets can be produced with high levels of hyperpolarization together with long magnetic state lifetimes using a variation of SABRE that they termed “SABRE-SHEATH.”^16,25–28^ It simply uses a mu-metal shield to enable efficient and direct polarization transfer from the hydride ligands of the catalyst to heteronuclei. A significant breakthrough was reflected in their studies of diazirines which were found to display both longitudinal magnetization and long lived singlet states after polarization transfer.^16^ ^15^N polarization levels of ∼5% were reported, and the associated singlet state had a lifetime of 23 min. This singlet state was revealed by the use of chemical asymmetry and built from the work by Levitt and co-workers who illustrated how long-lived singlet states (LLSs) sustain nuclear spin lifetimes beyond those of the normal *T*_1_ time scale through storage in disconnected Eigen states^29,30^ that are immune to the major mechanisms of relaxation.^31^ Examples of such systems have been found where these long-lived states have lifetimes that exceed 1 h, or 50 times the more usual *T*_1_ time scale, in room temperature solution.^32,33^

In this work, we use the SABRE variant SABRE-SHEATH to hyperpolarize two ^15^N_2_-based diazines and rationalize the basis of a simple route to their detection over long-time-scales. To broaden applicability, these agents were selected to represent two kinds of substrates that differ according to whether their coupled ^15^N-spins are chemically or magnetically different.

## II. EXPERIMENTAL METHODS AND RESULTS

The *p*-H_2_ used in this SABRE hyperpolarization study was created with more than 92% purity using an in-house *para-*hydrogen generator.^34^ Samples were prepared by mixing 5 mM of [IrCl(COD)(IMes)] (IMes = 1,3-bis(2,4,6-trimethylphenyl)imidazol-2-ylidene) with 30 mM of the substrate (**1** or **2** of Scheme 1) in 0.6 ml of methanol-*d*_4_ in a 5 mm NMR tube fitted with a J. Young Tap. After degassing using a freeze-pump-thaw method, the samples were activated by the introduction of H_2_ at a pressure of 3 bars. SABRE hyperpolarization experiments were then completed by filling the NMR tubes with *p*-H_2_ (3 bars) and subsequently shaking them vigorously in the specified magnetic field before detecting the resulting signal inside a high field NMR spectrometer (11.75 T). In these experiments, a mu-metal shield was used to reduce the background magnetic field to around 1000 times its normal value so that an mG top-up field can be applied to the sample through the application of a solenoid.^17^ The SABRE/SABRE-SHEATH hyperpolarization and sample transfer steps take place over 10–20 s.^35,36^ Since SABRE is reversible, sample re-hyperpolarization can be achieved within just a few seconds by repeating this procedure with fresh *p*-H_2_. In this way, accuracy and relaxation effects can readily be probed. The NMR measurements that feature in the final observation step were carried out at 298 K on an 11.75 T Bruker Avance III spectrometer using a TBI probe.

Pyridazine **1** contains a pair of coupled ^15^N spins that are chemically different. Earlier studies of several related pyridazine based substrates confirmed they can provide access to good ^1^H-SABRE hyperpolarization levels, thereby indicating the suitability of these systems.^27,28,37^ The hyperpolarization of 3,6-dichloropyridazine-^15^N_2_ has also been previously reported.^38^ The pyridazine motif is itself prevalent in a range of pharmacologically active agents, and hence, screening their NMR detection and magnetic state lifetimes is sensible.^39^

The chemical shift between the two inequivalent ^15^N sites in **1** was quantified to be 29.3 ppm (1485 Hz at 11.75 T), with a mutual spin-spin coupling of 23 Hz connecting them. During the SABRE process, **1** and a pair of *p*-H_2_ derived hydride ligands bind to the iridium catalyst ([Ir(H)_2_(NHC)(**1**)_3_]Cl) to temporarily create an AA’BC type 4-spin system at low-magnetic field where the *trans* hydride-^15^N coupling is around 20 Hz, the hydride-hydride coupling ∼−8 Hz, and the retained ^15^N-^15^N ∼|20| Hz. Consequently, the SABRE transfer mechanism for diazines **1** and **2** leads to direct population of the corresponding ^15^N_2_-spin system singlet state after dissociation.^16,18,26,37,40^ This is the result of the fact that the *J*_HH_ and *J*_NN_ couplings are sufficiently close in size to enable the ^15^N-singlet to become populated in fields where the difference in chemical shift between the two bound nitrogen resonances is smaller than that in the ^15^N-^15^N *J*-coupling.

**FIG. 1.**
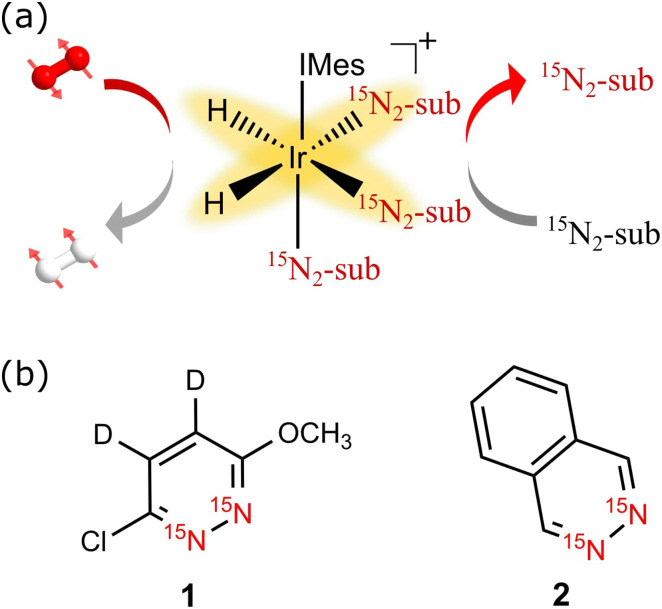
^15^N NMR spectra associated with **1**: single-shot hyperpolarized SABRE-SHEATH experiment detected at 11.75 T by a (a) 90° pulse and (b) 9° pulse; (c) the corresponding ^15^N NMR spectrum after 1000 averages.

Once SABRE hyperpolarization experiments were performed according to the aforementioned protocol in the case of **1**, adiabatic transfer to 11.75 T enables the observation of two ^15^N NMR signals, as detailed in Fig. 1(a), after a 90° hard observation pulse. These signals possess an “up-up-down-down” pattern that indirectly confirms the creation of ^15^N-singlet spin character in **1** after completion of SABRE.^17,41,42^ This is the result of probing a high-field state of the form *I*_z_*S*_z_ + *I*_z_ − *S*_z_ which leads to two observable doublets, of opposite relative phase, when interrogated by a 90° read pulse. When the same process was repeated, but using a 9° flip angle, the resulting NMR spectrum yields detectable outer-line transitions, as shown in Fig. 1(b), which further confirms the presence of initial singlet spin character as the *I*_z_*S*_z_ term which leads to a pair of antiphase doublets now adds to the earlier signal. These observations also show that the resulting state does not decohere rapidly which confirms that the presence of the methyl substituent has minimal effect on the signals’ lifetime.^43,44^ This is in agreement with the failure to observe any scalar coupling between the methyl group protons and the ^15^N centers. For comparison purposes, Fig. 1(c) shows the corresponding thermally polarized ^15^N NMR spectrum that was acquired in conjunction with the signal averaging over 1000 scans where the delay between measurements is 120 s. Consequently, this control measurement took over 33 h to make. On the basis of these data, a signal enhancement factor of 1250 could be determined at 11.75 T, relative to the thermally polarized NMR spectrum. These measurements were repeated using different polarization transfer field values in the range 1–10 mG, and little intensity variation was observed which is expected for a direct singlet transfer pathway.

Since the SABRE signal that is created in these experiments originates in the corresponding singlet state, its lifetime should be much longer than that associated with more usual *T*_1_ decay. Furthermore, since chemical shift anisotropy (CSA) is the major source of singlet order relaxation, this period should be extended significantly with lower magnetic field storage.^45^ This situation is complicated by the fact that the singlet (*S*_0_) state of the free material connects directly with the shorter lived triplet (*T*_o_) state which will act to reduce its population and therefore the high-field lifetime. However, when the substrate is bound, it will exhibit an even more reduced *S*_0_ lifetime due to the potential to transfer polarization into the hydride ligands in the reverse of the initial ^15^N polarization transfer step, and the existence of spin-spin couplings to the hydride ligands can also lead to the creation of triplet derived magnetization. These effects can be readily evaluated though by changing the metal concentration.

**FIG. 2.**
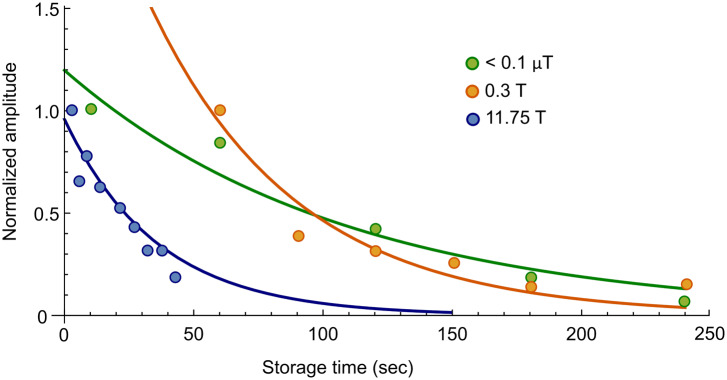
Normalized amplitudes of the ^15^N hyperpolarized NMR signals seen for **1** (circles) after SABRE-SHEATH as a function of sample storage time. Data points are fitted to an exponential (solid curves) which yields the signal lifetimes reported in Table I for a precatalyst to **1** loading of 1:6. Sample storage took place at 11.75 T (blue), 0.3 T (orange), and 0 T (green).

We therefore first measured the effective lifetime of the magnetization created under SABRE after storage in three magnetic fields. For the high magnetic field value, we used the 11.75 T field of the NMR system and determined the signal lifetime to be 35.8 ± 5.8 s. Next, the sample was examined after storage at 0.3 T, and the lifetime of the signal increased to 56.5 ± 12.6. The 0.3 T field was selected because of the work of Shchepin *et al*. where they found it proved suitable for hyperpolarized *T*_1_ extension.^46^ Upon storage in the mu-metal shield, the signal lifetime became 118.3 ± 20.4 s. The normalized signal intensities used in obtaining these values alongside the corresponding exponential fits are shown in Fig. 2, and the results are detailed in Table I. As indicated above, these signal lifetimes are each measured in the presence of the active SABRE catalyst. They are therefore further compressed by the reversible interaction of **1** with the catalyst which more efficiently breaks the symmetry of the magnetic state during the ligation event as δΔ increases to ∼3000 Hz for the ligand bound *trans* to hydride at 11.75 T. Consequently, when these measurements are repeated with a 50-fold excess of **1** based on iridium, rather than the 6-fold described first, these lifetimes are extended. Now, the signal lifetime becomes 48.8 ± 7.1 s at 11.75 T, while at zero field (mu-metal shield), it became 155.5 ± 15.4 s. Hence, we can conclude that the lifetimes can be substantially improved in the presence of a larger excess of the substrate which reduces the propensity for magnetization decay through ligand exchange. However, a significant drop in the signal enhancement factor to ∼200-fold is also observed at this higher substrate loading, and therefore, a balance between signal size and lifetime needs to be considered based on the desired application.

**TABLE I.** ^15^N polarization levels (enhancement factor and %) and signal lifetimes for **1** and **2** at the specified storage fields achieved with the precatalyst [IrCl(COD)(IMes)]. All measurements were made at 11.75 T and 298 K.

	Enhancement	Hyperpolarized	Hyperpolarized	Hyperpolarized signal
	factor (ε)and net	signal lifetime at	lifetime at 0.3 T	lifetime at 0.1 *µ*T
Agent	polarization (P%)	11.75 T	0.3 T	
**1**	ε: 1250	T_LLS_: 35.8 ± 5.8 s	T_LLS_: 56.5 ± 12.6 s	T_LLS_: 118.3 ± 20.4 s
	P: 0.5%			
**2**	ε: 4800	T_1_: 5.8 ± 0.2 s	T_1_: 56.0 ± 2.5 s	T_1_: 21.0 ± 6.3 s
	P: 2%			

Due to the long lifetime of the created hyperpolarized ^15^N signal of **1**, we could further improve the signal enhancement achieved with a sample containing 5 mM [IrCl(COD)(IMes)] and 30 mM of **1** by extending the polarization transfer times. When a 25 s polarization time was employed, the visible signal gain increased significantly to 2700-fold. This represents the detection of a signal that is twice as large as that achieved with a 10 s transfer time. However, when the polarization time was increased above 30 s, the ^15^N signal gain decreased to 2500-fold which reflects the finite volume of *p*-H_2_ that is present in the sealed NMR tubes used in this study.

The second substrate, phthalazine **2**, has a chemically equivalent but magnetically distinct ^15^N_2_-spin system due to the associated ^1^H couplings. It was probed under SABRE-SHEATH conditions inside a mu-metal shield as described above.^17,38^ The presence of the α-proton substituents on the ring system, and their visible couplings to ^15^N, will enable decoherence of any *S*_o_ term that is created through SABRE and thereby make the resulting states visible to NMR.^25,45^ However, as indicted earlier, the transient binding of **2** to a metal complex will break both the chemical and magnetic symmetry of this ^15^N pair, thereby providing not only a route to see both bound and free materials but also a route to further decohere the singlet state, in a process whose effect will again be concentration dependent.

**FIG. 3.**
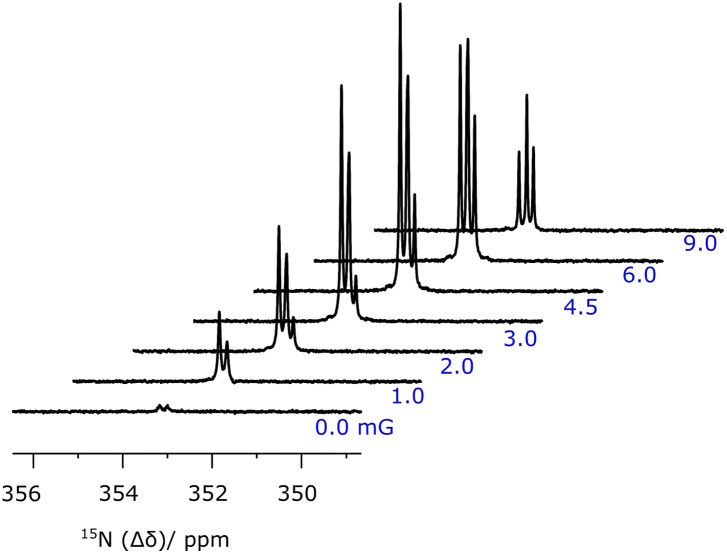
Hyperpolarized ^15^N NMR spectra of **2** as a function of the mixing field (0–9 mG) experienced during polarization transfer. The NMR tube sample was mixed with *p*-H_2_ inside a voltage-controlled coil that was placed inside a mu-metal shielded chamber for these measurements.

For these measurements, we initially employed a solution containing 5 mM of [IrCl(COD)(IMes)] and 30 mM of **2**. Figure 3 shows the resulting series of hyperpolarized ^15^N NMR signals for **2** that were observed after the application of a 90° observation pulse as a function of the polarization transfer field strength, and we see the signal reaches maximum amplitude at 4.5 mG.

The polarization of **2** is achieved through the creation of an initially identical AA’BC spin network on the catalyst as with **1**, where *J*_NN_ is now approximately 20 Hz and *J*_(*trans*-hydride)N_ = 16 Hz and *J*_(*cis*-hydride)N_ <1 Hz (neglecting the ^2^*J*_NH_ coupling to the α ring proton of 6.5 Hz within **2**). When **2** is bound *trans* to NHC, the associated ^15^N-hydride couplings are <1 Hz. Upon ligand dissociation, the singlet state in free **2** that is created under SABRE can only evolve under the smaller weak symmetry breaking α−proton-nitrogen spin-spin couplings (^2^*J*_NH_ = 6.5 Hz, ^3^*J*_NH_ < 1 Hz) and will have a longer lifetime than that in the bound material.

**FIG. 4.**
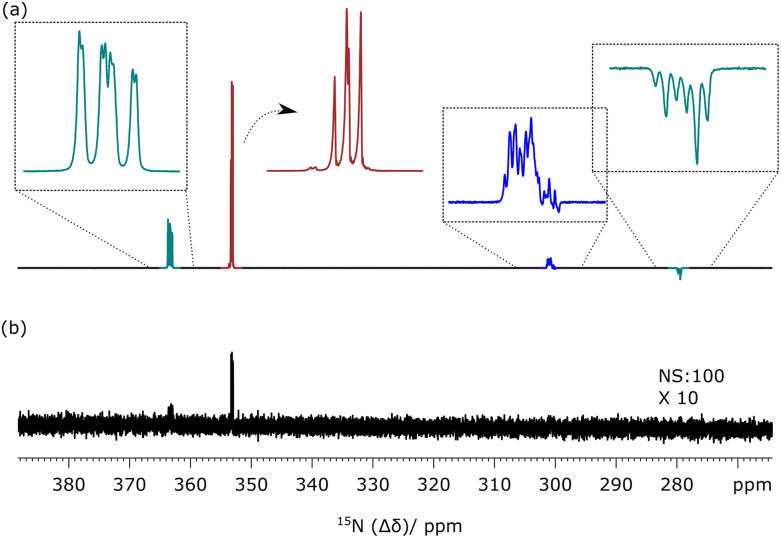
(a) High field single shot ^15^N NMR SABRE-SHEATH spectrum of **2** after polarization transfer at 4.5 mG. Expansions show the “free-**2**” peak at 353 ppm (red) and “bound” axial ligand peaks (green, dominant) and the equatorial ligand signals (blue for the bound nitrogen atoms) with characteristic singlet features. (b) ^15^N thermal polarized NMR spectrum using 100 transients that is vertically scaled by 10 compared to (a).

**FIG. 5.**
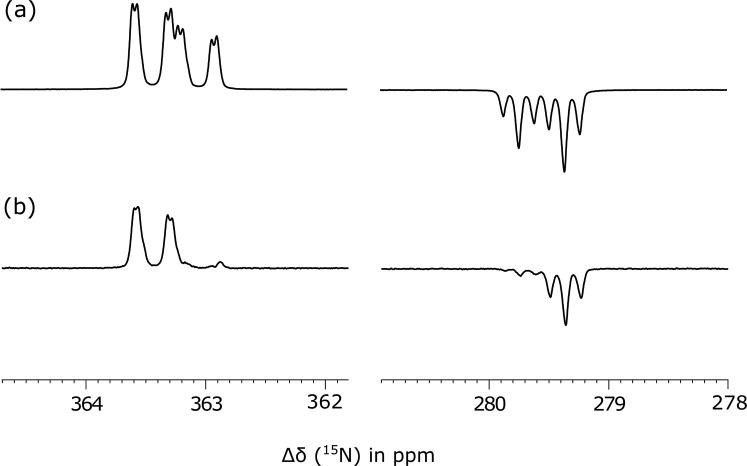
^15^N NMR spectra showing the axially bound ligand peaks of **2** [Ir(H)_2_(IMes)(**2**)_3_]Cl that are visible after SABRE-SHEATH and through (a) a 90° pulse and (b) a 9° pulse.

Figure 4 shows all of the detected ^15^N resonances after SABRE transfer at 4.5 mG where additional peaks due to bound **2** within this catalyst are clearly present. A similar “up-up-down-down” ^15^N NMR pattern is readily seen for the two ^15^N-coupled spins of **2** when it is located in *trans* to NHC (δ 362.8 and 278.8) in the SABRE catalyst [Ir(H)_2_(NHC)(**2**)_3_]Cl, as the additional symmetry breaking couplings to hydride are now much weaker**.** The peaks with significantly reduced amplitude correspond to the more rapidly exchanging equatorial-ligands (δ 302.6 and 299.7) that couple strongly to hydride and consequently relax more rapidly. Confirmation of singlet character in these probed states was again provided by small tip-angle pulse examination which leads to the detection of two outer transitions in all cases (Fig. 5). The process of substrate dissociation from the iridium catalyst returns to the symmetric ^15^N_2_-environment of **2** in these measurements, as proposed earlier, and thereby promotes further, albeit slower singlet state decoherence. The observation of these signals in bound **2** is therefore reflective of indirect confirmation that **2** was initially present in the singlet form.

The signal enhancements for the less sterically demanding **2** were significantly higher than those achieved for **1** under these SABRE conditions, and a ^15^N control signal [Fig. 4(b)] confirmed the enhancement factor was now 4800 at 11.75 T (∼2%). Changing the SABRE catalyst to a *tert*-butyl-substituted catalyst^47^ raised this level to 14 500-fold (∼6%) under similar conditions. Consequently, the lifetime over which the signal in the “bound” ligand remained visible was less than 10 s in accordance with a rapid ligand loss rate of ∼0.4 s^−1^ which leads to rapid cycling of this material.^37^

**FIG. 6.**
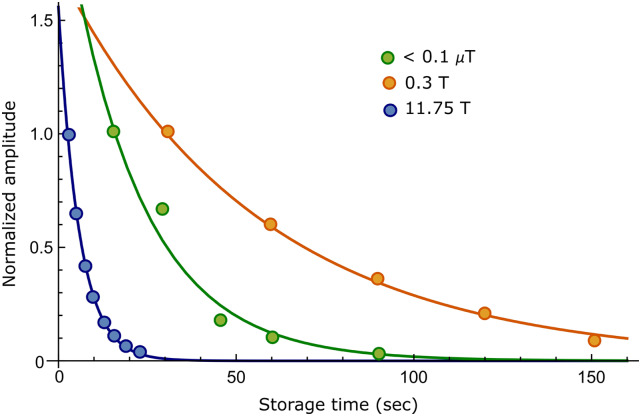
Normalized amplitude of ^15^N hyperpolarized NMR signals of **2** (circles) observed after SABRE SHEATH as a function of sample storage time. Data points were fitted to exponentials (solid curves), and the results are detailed in Table I. Three different magnetic storage fields were used: 11.75 T (blue), 0.3 T (orange), and 0 T (green).

The lifetime of the magnetism responsible for the signal of hyperpolarized **2** was then studied in more detail. Its high-field lifetime time proved to be 5.8 ± 0.2 s. A lifetime of 21.0 ± 6.3 s was then determined for storage in the mu-metal shield, while upon storage at 0.3 T, it became 56.0 ± 2.5 s. Figure 6 shows the normalized hyperpolarized signal amplitude observed for **2** in these three storage fields. Table I details the enhancement factor and lifetimes of **2**. These results are again affected by the catalyst and substrate concentration, and when a 50-fold excess of **2** when compared to the catalyst was utilized, these signal lifetimes were increased by ∼40%. This scale of change is similar to that of previous reports and is a consequence of the catalyst contribution to the singlet state decoherence being reduced, although the contribution of the intraligand H-^15^N coupling to signal decay remains.^27,43^

## III. CONCLUSION

In summary, we have reported how SABRE hyperpolarization can improve the ^15^N detectability of 3-chloro-6-methoxy-4,5-*d*_2_-pyridazine-^15^N_2_ (**1**) and phthalazine-^15^N_2_ (**2**). These molecules were synthesized as representative examples of pyridazine derivatives that possess a strong ^15^N-^15^N coupling (∼20 Hz). Consequently, we expected to be able to prepare them in a singlet state through low-field polarization transfer via an SABRE catalyst of the form [Ir(H)_2_(NHC)(sub)_3_]Cl where the associated hydride-hydride coupling will be of the order of −8 Hz. In the case of **1**, the steric bulk of the agent limits the efficiency of SABRE transfer such that a 0.5% polarization level is achieved; however, the isolated spin system exhibits an impressive NMR signal lifetime of 155 s when stored inside a mu metal shield. In the case of **2**, it is easier to achieve higher levels of hyperpolarization due to the reduced steric bulk of this agent. Consequently, when a *tert*-butyl-substituted precatalyst is employed, 6% ^15^N polarization is achieved. This hyperpolarization is readily read out by breaking the symmetry of the spin system of **2** through binding to the catalyst with the result that two strong inequivalent signals are detected in the associated ^15^N NMR responses of bound **2** when it lies *trans* to NHC in [Ir(H)_2_(NHC)(**2**)_3_]Cl. Again, rapid ligand exchange with the SABRE catalyst reduces the apparent signal lifetime to 75 s for a 50-fold excess of reagent at a 0.3 T storage field.^46^ This effect arises because ligand binding leads to a situation where δΔ for the two ^15^N sites increases from 0 Hz in free **2** to ∼4000 Hz when bound at 11.75 T, depending on the ligand geometry, while introducing a further J_HN_ coupling of ∼20 Hz when bound *trans* to hydride with J_HH_ = −8 Hz and J_NN_ ∼|20| Hz. These couplings and chemical shift changes enable the initially created singlet order to interconvert into the triplet manifold, thereby further reducing signal lifetime. This effect is substantial, leading to a 40% fall in signal lifetime on moving from a 50-fold to a 6-fold ligand excess. Although we expect further catalyst optimizations to dramatically increase these levels of ^15^N-signal gain, it will be important to remove the catalyst if the period over which a signal is to be detected is maximized. This will be especially true if *in vivo* ^15^N measurement is the aim.

## IV. METHODS

### A. ^15^N_2_-*d*_2_-maleic hydrazide

^15^N_2_-hydrazine sulfate (500 mg, 3.79 mmol, 1.0 eq) was added to a stirred solution of *d*_2_-maleic anhydride (500 mg, 5.0 mmol, 1.32 eq) in water (7 ml). The resulting solution was heated to 100 °C for 3 h before being allowed to cool to RT. The reaction was filtered, and the precipitate was collected and dried under reduced pressure to give ^15^N_2_-*d*_2_-maleic hydrazide as a white solid which was used in the next step without further purification.

### B. ^15^N_2_-3,6-dichloro-4,5-*d*_2_-pyridazine

^15^N_2_-*d*_2_-maleic hydrazide (325 mg, 2.80 mmol, 1.0 eq.) in POCl_3_ (3.0 ml) was heated to 95 °C for 3 h. Then, the reaction was cooled to RT and added dropwise to an ice cold solution of NaHCO_3_ to neutralize. EtOAc (15 ml) was added, and the two layers were separated. The aqueous layer was extracted with EtOAc (3 × 15 ml), and the combined organic layers were dried (MgSO_4_) and concentrated under reduced pressure to give the crude product. Purification by flash column chromatography with 8:2 hexane-EtOAc as the eluent gave ^15^N_2_-3,6-dichloro-4,5-*d*_2_-pyridazine (321 mg, 75%) as a white solid, with *R*_F_ (8:2 hexane-EtOAc) 0.3; ^**13**^**C NMR** (126 MHz, CDCl_3_): *δ* (ppm) 156.0 (t, *J =* 7.24 Hz) and 130.0 (t, *J* = 26.6 Hz); ^**15**^**N NMR** (51 MHz, CDCl_3_): *δ* (ppm) 390.2 (s); **MS** (ESI): *m/z* 175 [(M + Na)^+^, 40] and 153 [(M + H)^+^, 100]; **HRMS** (ESI): *m/z* [M + Na]^+^ calculated for C_4_Cl_2_D_2_^15^N_2_ 174.9553, found 174.9559 (−3.0 ppm error).

### C. ^15^N_2_-3-chloro- 4,5-*d*_2_-6-methoxypyridazine (**1**)

Sodium methoxide (60 mg, 1.1 mmol, 1.1 eq.) was added to a stirred solution of ^15^N_2_-3,6-dichloro-4,5-*d*_2_-pyridazine (153 mg, 1.0 mmol, 1.0 eq) in MeOH (10 ml), and the resulting solution was stirred at RT for 48 h. The reaction was concentrated under reduced pressure to give the crude product. Purification by flash column chromatography with 95:5-85:15 CH_2_Cl_2_-EtOAc as the eluent gave **1** (143 mg, 97%) as a white solid, with *R*_F_ (85:15 CH_2_Cl_2_-EtOAc) 0.3; ^**1**^**H NMR** (500 MHz, CDCl_3_): *δ* (ppm) 4.12 (s, 3H); ^**13**^**C NMR** (126 MHz, CDCl_3_): *δ* (ppm) 164.4 (d, *J* = 5.3 Hz), 151.0 (m), 130.3 (app. t, *J* = 24.3 Hz), 119.7 (dd, *J* = 23.4, 3.8 Hz), and 55.2 (d, *J* = 4.0 Hz); ^**15**^**N NMR** (41 MHz, CDCl_3_): *δ* (ppm) 372.3 (d, *J* = 23.7 Hz) and 339.9 (d, *J* = 23.7 Hz); **MS** (ESI): *m/z* 171 [(M + Na)^+^, 80] and 149 [(M + H)^+^, 100]; **HRMS** (ESI): *m/z* [M + Na]^+^ calculated for C_5_H_3_ClD_2_^15^N_2_O 171.0049, found 171.0053 (−1.7 ppm error).

### D. ^15^N_2_-phthalazine (**2**)

A solution of ^15^N_2_H_4_.H_2_SO_4_ (1.21 g, 9.31 mmol) in 1M NaOH (15 ml) was added to a solution of phthaldialdehyde (1.25 g, 9.33 mmol) and EtOH (30 ml) at room temperature and stirred for 3 h. The resulting solution was extracted with DCM (3 × 100 ml^2^) and the combined extracts concentrated *in vacuo*. Purification by column chromatography (EtOAc) afforded **2** (815 mg, 66%) as an orange solid. ^**1**^**H NMR** (400 MHz, CDCl_3_): δ (ppm) 9.43 (app. t, *J* = 8.2 Hz, 2H) and 7.87–7.81 (m, 4H); ^**13**^**C NMR** (101 MHz, CDCl_3_): δ (ppm) 151.1 (t, *J* = 4.4 Hz), 132.7, 126.4 (t, *J* = 1.8 Hz), and 126.2; ^**15**^**N NMR** (51 MHz, CDCl_3_): δ (ppm) 365.3; **MS** (ESI): *m/z* 155 [(M + Na)^+^, 100] and 133 [(M + H)^+^, 80]; **HRMS** (ESI): *m/z* [M + H]^+^ calculated for C_8_H_7_^15^N_2_ 133.0544, found 133.0548 (−2.5 ppm error).
